# Supplementary material for: Are carbon emissions trading and green financial instruments synergistic? -Comprehensive quantitative research based on content analysis
Source: PLoS One. 2024 Mar 7;19(3):e0298601. doi: 10.1371/journal.pone.0298601 (PMC10919601; doi:10.1371/journal.pone.0298601)
Supplement: S1 Data — (ZIP) [file pone.0298601.s001.zip › Data/地级市Z_F工作报告.zip!/░▓╗╒/╗┤▒▒╩╨/└┤╫╘╣1⁄2╓┌║┼í╛┬φ┐╦╩2╛▌═°í┐_2007.docx]

各位代表：

    现在，我代表市人民政府，向大会作工作报告，请予审议，并请各位政协委员和其他列席会议的同志提出意见。

                              ****一、去年的工作回顾****

    刚刚过去的2006年，市政府在市委的正确领导和市人大、市政协的监督支持下，团结和带领全市人民，坚持以科学发展观为统领，紧紧围绕市十三届人大四次、五次会议确定的目标任务，抢抓机遇、开拓进取，负重奋进、扎实工作，经济社会发展迈出了新的步伐。

     （一）国民经济平稳发展。适应宏观政策调控的新形势，抢抓中部崛起和省委省政府打造“两淮一蚌”重化工业走廊的新机遇，着力解决经济结构单一、前期投资不足等突出矛盾和问题，加快推进经济结构调整和增长方式转变，促进了国民经济发展速度加快、质量改善和效益提升。全年实现生产总值226亿元，增长10.1%；完成财政总收入31.2亿元，增长14%；城镇居民人均可支配收入9807元，增长14％；农民人均纯收入2968元，增长11.4%。

     工业经济平稳增长。积极推进工业强市战略，大力组织实施“3588”工业振兴计划，强化经济发展主动力。出台加快新型工业化进程等政策，实施优势资源转化、支柱产业带动、工业园区集聚等，市场主体培育取得成效，规模以上工业企业新增49家。切实强化运行调度，引导企业加强管理，规模以上工业企业完成增加值102.3亿元，增长8.1%；企业综合效益指数达120.9%，实现利税总额29.5亿元。支持质量兴企，鼓励创立名牌，3个产品获国家免检产品称号、3个产品被评为安徽省名牌。县区工业快速发展，增加值平均增长38%，高于全市30个百分点。

     农村经济健康发展。落实国家各项惠农政策，发放各种涉农补贴7621万元。大力实施区域化种植、规模化生产、产业化经营和社会化服务，小麦高产攻关单产全省第一，粮食总产再创历史新高；完成人工造林840公顷、绿色长廊二期工程73公里，林业加快发展；加大种猪、种禽场扶持力度，畜牧业恢复性增长；新增规模以上农产品加工龙头企业11家，实现销售收入15.6亿元；新型农村合作经济组织建设走在全省前列，农村能源建设切实加强。大力发展劳务经济，新增劳务输出3.3万人，实现劳务收入22亿元。启动实施“五镇十村”新农村建设示范工程，特别是农村基础设施建设得到加强，临涣闸、化家湖水库加固工程等投入使用，数万农民安全饮水问题得到解决，生产用水矛盾有所缓解；建成农村公路478公里，其中“村村通”工程完成386.3公里，位居全省前列；扎实推进农村电网改造，实现了城乡同网同价，支撑发展的能力进一步增强。

     第三产业快速壮大。围绕提高城乡居民生活水平，积极繁荣活跃消费市场，全社会消费品零售总额完成61.9亿元，增长13.9%，为近年来最好水平。大力推进“万村千乡工程”试点建设，努力构建城乡双向流通网络，新增农家店302个、配送中心4个，受到省政府通报表彰。专业市场建设步伐加快，矿业博览城已经启动，久兴（东岗楼）批发市场加快建设。建立重要商品储备和应急报告制度，加大市场运行监测和调控工作力度，3家企业被确定为国家级应急商品数据库重点联系企业，1家企业被商务部确定为国家（生猪活体）储备基地。千方百计扩大外贸进出口，完成进出口总额5361万美元，实现省、市调整目标。

    （二）有效投入持续增加。坚持有保有压，优化投资和项目结构，扎实推进重点产业基地和重大基础工程建设，一批事关全局发展的重大项目取得突破。固定资产投资高位攀升，促进了即期经济增长。完成固定资产投资110.4亿元，增长42.8%，其中制造业完成投资21.9亿元，增长99%；采掘业完成投资49.3亿元，增长29.8%；组织实施技术创新56项，技术改造投资完成13.8亿元。重点项目建设扎实推进，增强了持续发展后劲。临涣煤焦化电焦化工程一号焦炉投产，南坪煤化工项目继续推进。许疃、祁东等改扩建矿井即将竣工投产，涡北矿井进入联合试运转阶段，卧龙湖、五沟等在建矿井进展顺利；恒源、祁东煤泥矸石电厂进入试生产，50万伏变电站及电网改造项目加紧建设，国安电力二期、平山电厂工程前期工作扎实推进；辉克药业、龙波电器等一批非煤电高科技项目建设加快。按照“续建一批、开工一批、推进一批、储备一批”的思路，先后两次调整充实项目库，入库项目292个，总投资1083亿元。

    （三）改革开放成效显著。招商引资取得新突破。建立市级领导联系招商组及重点招商项目制度，开展驻点、离岗、企业和节会招商等，实际利用市外资金70亿元，增长108.6%；实际利用外资4532万美元，增长68.9%；新引进2000万元以上工业项目111个，新批外商投资企业21家。特别是围绕提高招商引资工作的针对性、选择性和实效性，成功举办首届中国（淮北）煤矿机械装备投资贸易洽谈会，签约合作项目62个，协议投资额27.4亿元。工业园区建设取得新突破。举全市之力加快市经济开发区建设，完成基础设施建设投入3亿元，龙湖工业园起步区发展框架已经形成；创新运行机制，实行全程代理服务，强化招商引资，引进资金10.7亿元，增长127.6%；引进1000万元以上大项目29个，增长163.6%。各县区工业园建设力度加大，特别是淮北杜集经济开发区比照省级开发区筹建进展顺利，10平方公里控制性规划已经完成，起步区实现“五通一平”，并有一批企业相继入驻。全民创业取得新突破。成功举办首届中国（淮北）专利技术推介会，签订合作合同、协议或意向227个，并首批成为全国18家获准建设“中国专利技术展示交易中心”的城市之一。制定和完善优惠政策，积极开展银企合作，加强创业基地建设，强化技能培训，引导和促进创业，新发展私营企业885家，新增个体工商户5363户，新增从业人员15877人。国企改革取得新突破。列入改制计划的163户市属国有、集体企业改制面达98.2%，改革步伐加快。淮北印染、相王啤酒、市燃气公司等一批企业引进了维科、华润等战略投资者，已转换机制、扩大生产，企业发展活力增强。多渠道筹措资金，累计支付改革成本5.3亿元，2万多名职工完成身份转换，1.6万名职工重新就业，职工利益得到较好维护。其他改革取得新突破。深化财政和投融资体制改革，硬化预算约束，加强非税收入管理，推行零余额账户管理；整合政府行政性资产和部分企业经营性资产，重组市国有资产运营公司，组建市信用担保公司，做大做强建投公司，共融资8.6亿元，支持了城市建设、开发区建设和企业改制。深化行政管理体制改革，理顺了一些部门的管理关系。深化农村综合改革，推进了兽医体制改革和林权、水权改革，乡镇政务服务中心建设步伐加快。

     （四）城市建设扎实推进。正确处理城市化与工业化、城市化与提高人民群众生活水平的互动关系，切实加大工作推进力度。城市规划进一步完善。《淮北市城市总体规划（2005—2020）》顺利通过国务院审批，《淮北市城市近期建设规划（2006—2010）》完成编制并批准实施，工业园区、住房建设、生态水景等专项规划编制完成，人民路东段和老城区等控制性详细规划正在编制。城市功能进一步提升。南湖中路、南黎东路、惠苑东路建成通车，惠黎路改造完成，董庄路、张寨路等加紧建设，全年新增市政道路22公里。老濉河改造等工程竣工，供水管网改造及区域供水工程完成，中水回用、医疗废弃物处置等工程加快建设，天然气、集中供热、垃圾处理等项目前期工作扎实推进。“城中村”改造稳步实施，房地产市场健康发展，新开工建设房地产85.3万平方米，增长39%；完成投资12.5亿元，增长41.7%。建成“爱园”等一批园林景观，培育一批园林式单位，新增园林绿地6.36公顷，完成相山造林绿化200公顷，建成区绿化覆盖率达39.3%，荣获“国家园林城市”称号。采煤沉陷区环境综合治理项目获“中国人居环境范例奖”，相山区被评为省环保模范城区。城市管理进一步加强。实施“四级卫生督查制度”，推行“城管110”受理服务，建设城管电子监控中心，开展占道经营、占道停车等专项整治和文明示范街创建活动，市容管理更加规范。

     （五）社会事业协调发展。高度重视民生问题。出台并落实《关于大力实施六大民生工程，积极构建和谐淮北的意见》。加强就业再就业工作，全年新增城镇就业岗位3.3万个，8626名下岗失业人员实现再就业。进一步提高城镇低保补差标准、企业退休人员待遇和城市居民最低工资标准，全年增加支付社会保险金6000万元。在全省率先建立农村居民最低生活保障制度，3万名特困群众及时纳入低保范围。积极推进新型农村合作医疗，濉溪县参合率达到89.6%，三区参合工作启动有力；市公共卫生大厦投入使用，城市社区卫生服务走在全省前列。经济适用房建设稳步推进，“南黎花园”一期项目即将竣工，946户城镇中低收入家庭即将搬入新居；启动廉租住房保障，保障户近期可领到住房补助。市社会福利中心建设筹备工作进展顺利，新建和扩建12个乡镇敬老院，社会救助工作继续走在全国先进行列。

     高度重视安全稳定。“五五”普法扎实推进，人民调解工作进一步加强。制定和完善75项总体、专项和部门突发公共事件预案，应急处置能力得到加强。深入开展综合治理，健全治安防控体系，严厉打击刑事犯罪，社会治安形势稳定。落实责任制和责任追究制，强化煤矿、非煤矿山等领域的监管和整治，安全生产形势稳定好转。全面贯彻新的《信访条例》，认真落实市领导接访制度，坚持以人为本，顺利完成久兴（东岗楼）批发市场停业搬迁，妥善化解了成品油价格上涨对公交车、出租汽车行业的影响，人民群众反映的一些热点难点问题得到解决。

     高度重视社会事业发展。科技创新步伐不断加快，依靠科技进步推动经济发展的成效显现、氛围形成。落实教育事业优先发展政策，改造农村中小学D级危房2万多平方米，新建校舍2.2万平方米，一批薄弱学校建设得到加强；市十二中、煤师院附中顺利跨入省示范高中行列，全市中小学整体办学水平跻身全省前列，职业教育工作受到省政府表彰。文化事业加快发展，创作生产了电视连续剧《大嫂》、电影《美丽的村庄》，农村无线微波数字电视系统开通，市广电中心开工建设，市新图书馆选址等前期工作进展顺利。体育事业平稳推进，成功承办了省第五届少数民族传统体育运动会，新体育场加快建设。群众性精神文明创建扎实推进，文明城市创建工作取得成效。认真贯彻落实国家调控政策，土地及矿产资源开发市场秩序得到规范，土地出让金清欠工作成效显著。建成市环境监控中心，大力开展小水泥等专项整治，环境保护得到加强。农村计生工作水平稳中有升，城市计生工作在全省继续保持领先。物价工作成效显著，全省综合评比第一。支持驻淮部队和武警部队建设，加强国防动员和后备力量建设，积极做好人民防空工作，军政、军民关系进一步密切。第二次全国农业普查工作进展顺利，统计工作水平不断提高。老龄、残疾人和妇女儿童等工作扎实推进，人事编制、民族宗教、外事侨务、对台事务、邮政通讯、新闻出版、公积金管理、机关事务管理、接待、气象、档案、地震、地方志等工作取得新进展。

     （六）行政效能不断提高。扎实开展“解放思想、提高效能”主题实践活动，深化行政审批制度改革，开展“三清理、三集中”，取消、下放、合并了一批行政审批事项，直接减少企业和社会负担4000多万元。切实强化市行政服务中心职能，认真落实5日办结制、首问负责制和责任追究制，“一站式”服务水平和办事效率明显提高。全面推行县级干部引咎辞职制、一次投诉查实离岗制、机关评议末位淘汰制等制度，发展环境不断优化。自觉接受人大法律监督、工作监督和政协民主监督，办理人大议案3件，代表建议130件，政协委员提案204件，搞好“投诉热线”和“行风热线”受理，开通市长热线电话，注意听取社会各界的意见和建议，群众利益得到较好维护。加强经济责任审计，加大对商业贿赂和损害群众利益不正之风的 治理力度，深入开展廉政建设和反腐败斗争，维护了政府形象。

     各位代表，成绩来之不易，这是省委、省政府和市委正确领导的结果，是市人大、市政协和社会各界关心支持的结果，是全市上下奋发努力、共同拼搏的结果。在此，我代表市人民政府，向全市广大工人、农民、知识分子和离退休老同志，向驻淮部队、武警官兵和政法干警，向各民主党派、人民团体和其他各界人士，致以崇高的敬意！向所有支持、关心淮北发展的同志们、朋友们，向在我市创业的境内外投资者、建设者，表示衷心的感谢！

     在肯定成绩的同时，我们也清醒地看到，淮北的发展仍然存在不少困难和问题。一是加快发展的基础还不稳固，经济结构单一，传统主导产业的技术含量不高和盈利能力下降，很大程度上决定了经济发展的近期走势；虽然接续产业和非煤产业在加快崛起、园区功能在逐步显现、全民创业和县域经济在快速发展，但实现量的扩张和质的飞跃还有一个过程。二是影响社会和谐的因素仍然不少，财政收支矛盾突出，社会建设和公共服务投入还不能满足需要；市场整顿、征地拆迁中侵害群众利益的问题还时有发生，全社会权责意识、法制环境还有待进一步增强和改善。三是政府职能转变还不适应形势发展的需要，少数机关重审批轻服务、重安排轻落实的问题依然存在，少数单位效率不高、服务不优的问题依然存在，少数干部素质较低、为政不廉的问题依然存在。对此，我们将认真对待，切实加以解决。

                              ****二、今年的主要任务****

     2007年，既是推进“实力、活力、魅力、和谐”淮北建设的重要一年，也是实现经济社会又好又快发展的关键之年。实现又好又快发展，既有机遇，也有挑战。一是宏观经济环境比较有利。宏观调控的立足点是提高经济运行质量和效益，保持平稳较快增长，促进速度、质量、效益相协调；今年全国经济将继续保持较快增长，能源刚性需求会继续保持较高水平，为我市煤电产业的发展提供了广阔空间。宏观调控的重点是调整投资与消费、内需与外需的关系，促进投资、出口、消费相协调；国家支持能源、原材料和加工制造业基地建设，支持资源型城市转型和新农村建设，省委、省政府着力打造沿淮城市群和“两淮一蚌”重化工业走廊，有利于我市做大做强主导产业，培育发展新兴产业，加快新农村建设；国家鼓励和支持东部地区梯度转移产业，有利于我市扩大招商引资，承接产业和资本转移；国家强化内需对经济增长的拉动作用，有利于我市发展、壮大和提升三产。宏观调控的主控点是控制能源消耗、环境污染和低层次重复建设，增强控制性指标以及项目开工条件的刚性约束，促进人口、资源、环境相协调，也与我市加快发展的产业和方向相一致。二是自身发展具备一定基础。发展思路进一步理清，已在全社会形成共识，并正在化为自觉行动；发展趋势进一步向好，扩大投资积聚的能量开始释放，一些新的增长点逐步形成；发展条件进一步改善，关系全局发展的部分重点工作取得突破，一批基础设施建设增强了经济社会发展的支撑能力。当然，随着宏观调控政策的到位，信贷、环境等约束趋紧，项目审批门槛提高，对新上投资项目的要求也会越来越高。对此，我们必须努力把政策研究得深入一些，把问题琢磨得透彻一些，把措施考虑得周全一些，既保持经济平稳较快增长，防止大的起落，又坚持好中求快、优中求进，更加注重发展质量、社会和谐和管理创新，在落实宏观调控中推动转型发展，在深化改革开放中推动创新发展，在统筹兼顾中推动协调发展，在解决民生问题中推动和谐发展。

     今年政府工作的指导思想是：****认真贯彻党的十六届六中全会、中央经济工作会议和市第六次党代会精神，全面落实科学发展观，深入实施“六大战略”，优化经济结构，转变增长方式，推进改革创新，扩大对外开放，促进社会和谐，努力实现经济社会又好又快地发展。****

     全市经济社会发展的主要预期目标是：地区生产总值增长12%，财政收入增长12%以上，固定资产投资增长30%以上，社会消费品零售总额增长13%，城镇居民人均可支配收入增长10%以上，农民人均纯收入增长7%，城镇登记失业率控制在4%以内，人口自然增长率控制在省下达指标以内，单位生产总值能耗下降4%，主要污染物排放量减少2%。

     为实现上述目标，将重点抓好八个方面的工作：

     （一）坚定不移地实施“3588”工业振兴计划，强力推进工业强市。通过投资和项目拉动，加快产业基地建设和产业结构调整；通过引导扶持，促进企业发展和即期增长。

     致力于形成“三足鼎立”经济结构，增加有效投入。适应项目建设从严管理要求，着力建设具有核心竞争力的产业项目。围绕做大做强煤电产业，竣工投产卧龙湖、涡北等矿井，续建孙疃、杨柳、刘店、青东等矿井，开工建设钱营孜煤矿、卧龙湖选煤厂等项目；积极推进国安电力二期、临涣煤矸石电厂和大唐发电机组技改项目，竣工50万伏输变电工程。围绕培育发展煤化工产业，竣工临涣煤焦化电焦化一期工程和取供水工程，开工建设临涣煤焦化电焦化二期和南坪煤化工项目。围绕发展先进制造业和高新技术产业，加快发展载能工业和非煤工业，建成并投产华润20万千升雪花啤酒扩建、淮北中安机械设备制造一期、纤维素生物质液体燃料中试基地等项目，续建烈山1亿平方米高档墙地陶瓷砖等项目，开工建设东磁铝业7条化成箔生产线等项目，力争全年完成工业投资100亿元。

     致力于加速壮大企业群体，增加规模企业总量。支持各类企业多形式、多渠道增加投入，扩张规模，增强实力。对优势企业实施优化升级，加大政策扶持和品牌提升力度，培植一批大企业集团。对劣势企业实施创新培育，加快技术改造和重组再生，淘汰落后生产能力，促进上规模、上水平。对中小企业实施促进提升，着力解决市场准入、人才引进、投融资等问题，推动制度、技术和管理创新，促进规模化、集群式发展。同时，加大对县区工业发展考核和扶持力度，积极培育成长性企业，确保全市新增规模以上工业企业100家。

     致力于促进经济即期增长，抓好现有企业生产经营。督促和引导行业加强管理，重点抓好纺织等工业企业的节本增效和扭亏增盈。密切关注煤电运等生产要素供应和市场行情变化，加强骨干企业和主导产业运行调度，推动一批有市场、效益好的企业满负荷生产，确保规模企业增加值两位数以上增长。

     （二）坚定不移地推进农民增收，全面加强新农村建设。落实工业反哺农业、城市支持农村和多予少取放活方针，着力从产业、规划、政策、制度等多层面统筹解决“三农”问题。

     积极发展现代农业。大力发展优质高产高效农业，继续组织实施百万亩小麦高产攻关活动，扶持抓好瓜菜、花卉等高效经济作物小区和石榴、林果等林业基地建设。把养殖业作为支柱产业来抓，继续加强肉猪等6大养殖基地建设，新建30个规模养殖小区。加快推进产业化经营，以“212”强龙工程为抓手，加大培育和引进力度，整合做大现有农产品加工龙头企业，引进一批大企业大集团来我市建基地办企业，延长产业链、提高附加值。建立健全服务体系，强化农业技术推广、动植物疫病防治、农业信息等服务，提高农民组织化程度。积极推进农产品质量安全和标准化体系建设，完成市级农产品检测中心资格认证工作。

     积极促进农民增收。推进金融和产业资本向农村转移，城市产业向农村延伸，大力发展二、三产业，拓宽农民增收渠道。完善城乡统一就业体制，加快农村劳动力转移，鼓励民工返乡创业，力争就地和转移就业新增2.2万人。落实惠农政策，探索建立农民负担监督管理长效机制，积极化解乡村债务，以减负促增收。
     积极建设新农村。制定新农村建设规划，引导基础设施、建设布局、产业发展、社会事业协调发展。充分发挥城乡交错、矿镇伴生的优势，重点抓好城郊改造提升、采煤沉陷搬迁重建、生态农业开发观光、矿村共建和村庄整治五种类型的新农村示范工程建设。加强农村基础设施建设，强化改水改厕，推进村容村貌整治，改善农村生活环境；强化农田水利化、机械化和信息化建设，重点完成8万亩标准农田和15万人安全饮水工程，提高农业设施装备水平。强化精神文明、农村政策、职业技能等教育，培育新型农民。全面推进农村综合改革，加快建立基层行政管理新框架，提高乡镇社会管理和公共服务能力。

     （三）坚定不移地扩大消费需求，大力发展第三产业。围绕强化经济增长的支撑力和提高人民群众的生活质量，适应产业结构和消费结构升级趋势，促进服务业发展。

     加快发展生产性服务业。制定和完善物流产业发展规划，畅通综合交通运输网络，推进物流体系与产业布局对接，重点加快“淮北市粮食物流中心”、矿业博览城、久兴（东岗楼）批发购物等一批区域性物流中心、物流园区和特色商品集散地建设。出台促进服务业发展政策措施，大力发展金融、保险、中介、咨询、产权交易、信息等现代服务业。

     加快发展消费性服务业。完善消费促进政策，扩大消费信贷规模和品种，培育消费热点。继续大力发展商贸业，建设老城区淮海路和新城区商业步行街两个城市商业中心，培育壮大新惠康、顺达等流通企业，争取沃尔玛等世界知名企业尽快落户淮北，发展连锁经营等新型业态，提升服务业的档次和水平。加大农村市场开拓力度，完成农村商品流通改革和市场建设试点任务，确保每个乡镇和50%以上的行政村建有农家店。扎实推进“社区商业双进”工程建设，大力发展家政、修理、保健等服务，增强社区商业功能，满足居民多样化需求。加强红色旅游、南湖湿地公园等项目建设，发展旅游经济。

     （四）坚定不移地实施东向发展，提高对外开放水平。坚持互利共赢的开放战略，响应省委、省政府“扩大开放年”系列活动，加强对外交流与合作，增强加快发展的外源动力。

     进一步提高招商引资的质量和水平。整合招商资源，组建专业、产业招商队伍，集中力量主攻重点城市和重大项目。发挥大企业主体和园区载体作用，主动承接沿海发达地区先进制造业、高新技术产业和现代服务业转移，加大煤化工、煤矿机械、新型建材、农产品加工等招商力度，引进技术含量高、产业带动强的大项目，促进产业结构升级。走市场带动产业发展之路，重点办好第二届中国（淮北）煤矿机械装备投资贸易洽谈会，推进煤机产业发展壮大。坚持“重点客户追踪，重点项目跟进”原则，力争全年引进内资100亿元、外资8000万美元。

     进一步提高园区建设的质量和水平。坚持要素集聚和错位发展原则，引导工业加速向园区集中，加快形成各具特色的块状优势产业集群。继续加强市经济开发区建设，完善配套8平方公里起步区；着力解决规划、土地、融资等问题，开工建设二期基础设施，加大招商引资和产业发展力度，确保工业生产总值、实际利用市外资金和园区建设规模在2006年的基础上翻一番。

     进一步提高对外贸易的质量和水平。认真落实国家和省出口退税、外贸研发基金等政策，积极培育壮大淮北维科印染等一批出口龙头企业，优化对外贸易结构，转变外贸增长方式。

     （五）坚定不移地推进各项改革，打造体制机制优势。正确处理改革发展稳定的关系，稳步务实地推进重点领域和关键环节改革。

     深入推进国企改革。以国有资本退出和职工身份转换为重点，切实加强对纺织一厂、针织一厂、纺织品公司以及部分商贸二级法人企业的改制指导，全力做好国企改革的扫尾工作。对已改制企业，继续深化改革，加快转换经营机制，建立新型劳资关系，扩大生产规模，提高综合竞争能力。进一步加强国有资产监督管理，严格产权交易和股权转让秩序，防止国有资产流失；完善国有资本经营制度，促进国有资产保值增值。

     深入推进重点领域改革。一是继续推进财政体制改革，围绕增强政府调控能力、集中财力办大事，强化预算管理和非税收入管理，提高公共财政水平；理顺市、县（区）财税分配关系，扩大县区经济管理权限。二是加大投融资体制改革力度，整合做大各投融资公司，支持经济建设和社会建设。三是积极稳妥地推进事业单位改革，重点推进经营服务类事业单位转企改制。

     深入推进全民创业。全面落实扶持非公有制经济发展的政策措施，筹办好第二届中国（淮北）专利技术推介会，积极搭建银企合作、信用担保、信息网络和人才培训等平台，建立市级创业园区，开展社会化服务，激发加快发展的内生动力，力争新增私营企业1000家，新增个体工商户6000户。

     （六）坚定不移地统筹城乡发展，建设双百双宜城市。以国务院批准的城市总体规划为统领，呼应省委、省政府启动城市群规划建设，着力提高城市综合承载能力，强化城镇的规模效应、聚集效应和辐射效应，促进协调发展。

     加快推进双百双宜城市建设。围绕提高规划引领发展的水平，完成城市住房建设和老城区控制性详规等规划编制，抓好城市总体规划和近期规划实施。围绕提高经济社会发展支撑能力，加快基础设施建设，改造市区道路、老城区街道，续建南湖北路、张寨路、董庄路、梅苑路；坚持公交优先，积极推进场站建设；竣工污水处理二期、中水回用等工程，加快推进天然气利用工程和集中供热工程建设；开工建设跃进河公园、矿山公园等项目，抓好相山第五期绿化，巩固和提升园林城市成果；加快建设闸河三期工程，完成龙河开发区段治理，沟通城市水系，强化供水节水管理，确保城市水安全。围绕提高城市管理水平，完善相对集中行政处罚权制度，实施城市网格化、精细化和法治化管理。

     加快推进城镇化建设。加强县城规划建设管理与主城区的衔接，扎实推进市县一体化。落实扶持政策，支持县区围绕重大项目和重点城镇建设发展配套，促进人口与产业集聚。加强交通基础设施建设，重点配合市煤化工项目和工业园区发展，拓宽改造S202淮北段25公里，改建濉永路县道8.6公里；再建农村“村村通”公路266公里，配套发展农村班线，改善县域发展条件。

     加快推进市矿统筹建设。尽快编制市矿统筹各项规划，推动市矿资源共享、基础设施共建、产业协调发展。优化矿区投资环境，保障重点工程建设；加强市矿协作，妥善解决矿区采煤沉陷征地迁村、职工住房建设、城市建设发展压覆资源、征地款拖欠等问题。抓好临涣和南坪两个煤化工园区规划与控制，高起点建设淮北市南部新区。

     （七）坚定不移地关注民生，努力构建和谐淮北。坚持尽力而为、量力而行原则，着力构筑多层次、高质量、普惠型的社会支撑体系，促进人的全面发展。

     大力实施民生工程。一是全面落实税费减免、岗位补贴等优惠政策，高度重视大中专毕业生、复转军人安置，统筹做好城镇新增劳动力、被征地农民和农村富余劳动力转移就业的服务工作，加强劳动力市场建设，力争新增城镇就业岗位3.3万个。二是加强社会保险扩面征缴工作，认真做好城镇居民医疗保险、未参保企业退休人员参加医疗保险工作，探索建立健全被征地农民养老保险制度。三是推动义务教育均衡发展，鼓励发展民办教育，大力加强素质教育，加快发展职业教育和高等教育；高度关注农村中小学教育，全面消除农村中小学D级危房，积极推进中小学现代远程教育，选聘一批本科、大专毕业生到农村学校任教，改善农村办学条件，提高教学质量；积极推进梅苑学校等中小学建设，改扩建部分市区中小学校，缓解主城区教育资源不足的矛盾。四是继续加强公共卫生体系建设，强化疾病预防控制，提升应急处理水平；全面实施新型农村合作医疗，加强城市社区卫生服务，选派100名城区医院医务人员下乡帮扶，方便城乡群众防病治病；继续实行特困人群住院费用减免政策，完善城乡大病医疗救助制度。五是重点发展居民自住中低价位、中小套型的普通商住房，以及面向中低收入家庭的保障性住房，新建20万平方米经济适用房；继续实行廉租住房租金补贴保障，逐步解决低收入家庭住房困难；合理控制拆迁规模，稳步推进危旧房改造。六是完善城市低保制度和社会救助制度，推进分类施保，加强农村“五保”供养工作，加快市社会福利中心和市残疾人综合服务基地建设，加快发展慈善事业。

     大力发展社会事业。深入开展群众性精神文明创建活动，切实抓好公民特别是青少年思想品德和法制教育，加强科普宣传，倡导科学、健康、文明的生活方式，努力争创全省首批文明城市，进一步夯实创建全国文明城市的工作基础。积极推进文化体制改革，不断加强农村和城市社区文化建设，抓好文化、出版物市场管理，续建市广电大楼，开工建设市图书馆，推进广播电视“村村通”工程，做好全国第三次文物普查工作。深入实施全民健身行动计划，办好市第八届体育运动会，建成新体育场。加强基层基础工作，稳定低生育水平，提高出生人口素质，降低出生人口性别比。加强国防后备力量建设，做好人民防空、民兵预备役和拥军优属工作。依法维护妇女儿童合法权益。抓好第二次全国农业普查。继续做好人事编制、民族宗教、外事侨务、气象、档案、防震减灾、机关事务管理、接待、地方志等工作。

     大力建设平安淮北。深入实施“五五”普法，改进法律援助，加强人民调解工作。强化社会治安综合治理，搞好社区、农村、校园等警务建设，完善治安防控体系，依法打击“法轮功”邪教组织和各类违法犯罪活动。落实信访工作责任制，坚持领导带头接访、带案下访制度，有效预防和妥善处置群体性事件。坚持“预防为主、综合治理”方针，着力构建以科技为支撑、制度为基础、监管为手段的安全生产保障体系，严防重特大事故发生。

     （八）坚定不移地优化要素配置，增强可持续发展能力。发挥自身优势，消除“瓶颈”制约，在增强自主创新能力、生产要素供给和生态环境等方面迈出实质性步伐。提高创新能力。围绕建设创新型城市，充分发挥企业自主创新的主体作用，支持企业增加研发投入；切实加快专利技术展示交易中心、煤化工与精细化工数据库和科技孵化器建设，加强产学研结合，提高科技型中小企业培育和科技成果交易、转化、应用能力。发挥电力优势。加强电力生产企业富裕电量消化利用研究，协调推进电源点建设、电价调整和产业发展，变电力优势为经济优势。节约集约用地。整顿和规范资源开发秩序，落实基本农田保护措施，突出抓好采煤沉陷地开发利用，努力满足发展和建设用地需求。抓好银企协作。继续引导银行切实加大有效信贷投放力度，尤其是对中小企业的投入，提高存贷比；教育企业加强信用建设，依法诚信经营；规范运作运营公司、担保公司，为企业提供担保服务，为银行分担金融风险。建设资源节约型和环境友好型社会。围绕资源节约，建立健全节能管理和促进机制，突出抓好化工、建材、酿酒、纺织等行业的节能技术改造，加快构建节能型产业结构；大力发展循环经济，加快建立循环经济型企业和工业园区，争取列入国家循环经济试点市。围绕环境友好，加快建立生态环境评价体系和补偿机制，强化小水泥等专项整治和辐射源、危险及医疗废弃物监管，严格查处超标排污企业和未经环评项目，启动建设生态市，创建国家环保模范城；开工建设淮水北调工程，全面推进节水型社会、节水型城市试点建设和采煤沉陷区水资源综合利用；扎实推进创建全国绿化模范城活动，继续搞好荒山造林、退耕还林等城乡绿化。

                              ****三、切实加强政府自身建设****

    实现又好又快发展，建设文明和谐淮北，对政府工作提出了新的更高的要求。我们将牢固树立科学发展观和正确政绩观，以“创新促发展、执行看落实”为主题，扎实推进管理创新，努力打造顺应时代潮流、体现人民意志的现代政府。

     （一）建设以人为本、为民服务的政府。把尊重群众意愿与发展群众利益结合起来，不提不切实际的目标和口号，不搞劳民伤财的“政绩工程”和“形象工程”，多做打基础、增后劲、管长远、惠百姓的工作。抓好市长热线、政府网站建设，建立政府新闻发言人制度，完善政风、行风评议制度，并把人民群众关心的事项和关注的领域作为政务公开的重点，切实保障人民群众的知情权、参与权、选择权和监督权。转变政府职能，更加注重社会管理和公共服务，加快推进政资、政事分开，政府与市场中介组织分开，把该管的管住管好，不该管的交给企业、中介组织和市场；加快建立健全突发公共事件预警和应急处理机制，提高应急处理能力和抗风险能力；扎实开展食品、药品等专项治理，加强价格监测和预警以及房地产市场监管，规范市场秩序；以发展社会事业和解决民生问题为重点，进一步优化财政支出结构，把更多的财力投入公共建设。注意兼顾各方利益，照顾各方关切，把握好改革的力度、发展的速度和群众可以承受的程度，既在加快发展上创造新业绩，又在和谐发展中探索新路径，让更多的改革发展成果惠及百姓。

     （二）建设行为规范、依法行政的政府。把依法行政贯穿于政府决策、执行、监督的全过程，切实把政府管理纳入依法运转的轨道。自觉贯彻落实市委的各项部署和决定，依法接受人大及其常委会的监督，积极接受政协监督，认真办好人大议案、代表建议和政协委员提案，支持民主党派、工商联和人民团体依照章程开展工作，高度重视和支持人民群众与新闻舆论监督。建立健全重大问题集体决策、专家咨询、社会公示听证、决策效果评估制度，提升科学化民主化水平。规范行政执法行为，促进严格执法、公正执法、文明执法，保证各级行政管理机关及其工作人员严格按照法定权限和程序行使职权、履行职责。加强规范性文件合法性审查，注重通过行政复议等法定渠道预防和化解行政争议。严格落实行政执法责任制和执法过错追究制，深入推进相对集中行政处罚权，坚决克服多头执法、执法不公、执法违法的现象。

     （三）建设管理创新、务实高效的政府。把创新管理与优质服务结合起来，改革政府管理体制，理顺权责关系，找准上下之间、条块之间的“结合点”和行业之间的“共同点”，强化协调联动，形成推动工作的整体合力。继续深化行政审批制度改革，减少和规范行政审批事项，探索相对集中行政审批权，把工作重心从前置性审批转向后续监督。继续加强行政服务中心建设，落实“首席代表制”，推进“两集中”、“两到位”，加大并联审批力度，进一步提高办事效率。切实加强绩效管理、目标管理和效能建设，对已明确的任务、确定的事项，落实分工负责制、过程督查制、结果追问制，确保目标分解到位、压力传递到位、工作落实到位；对群众关注的热点、经济社会生活中的难点、实际工作中的薄弱点，拿出切实有效措施，高标准地做好每一项工作，高效率地解决好每一个问题，必要时杀几个“回马枪”，来几次“回头看”，力求更加务实、落实，不断增强政府的执行力和公信力。转变机关作风，减少文山会海，引导和激励广大公务员凝心聚力不分神，埋头苦干不浮躁，多做一些深入实际的调查研究，多解决一些影响改革发展稳定的突出问题，多办一些为广大人民群众排扰解难的实事。

     （四）建设纪律严明、清正廉洁的政府。坚持标本兼治、综合治理、惩防并举方针，全面落实廉政建设和反腐败工作责任制。从政府班子做起，牢记“两个务必”，带头发扬艰苦奋斗、勤俭节约的精神，带头反对铺张浪费和大手大脚，带头抵制拜金主义和奢糜之风，做到生活正派、情趣健康，讲操守、重品行。切实加强公务员队伍的教育、培训和管理，全面提高政治素质、理论素质和业务素质，不断增强工作推进能力和拒腐防变能力，努力做到有本事、能干事、干成事、不出事。强化行政监察，切实解决好条块分割、权责脱节、推诿扯皮，行政不作为和乱作为，以及有人出题、无人作文的问题，严肃查处干扰和破坏发展环境的行为。以改革和制度建设为重点，努力形成用制度管权、按制度办事、靠制度管人的有效机制，强化对权力运行的制约和监督，从源头上预防和治理腐败。以与群众利益密切相关的公共部门、单位为重点，认真抓好大案要案的查处，切实纠正损害群众利益的不正之风，严厉打击工程建设、土地出让、产权交易、医药购销、政府采购、资源开发与经销等重点领域的腐败行为，努力做廉洁勤政、执政为民的政府。

    各位代表！新的目标令人鼓舞，新的形势催人奋进。让我们在市委的坚强领导下，全面落实科学发展观，紧紧团结和依靠全市人民，振奋精神、团结拼搏，创新创业、跨越发展，为圆满完成全年各项目标任务，加快建设“更具实力、充满活力、富有魅力、文明和谐”的现代化工业城市而努力奋斗！
